# A proposed new rotating reference axis for the tibial component after proximal tibial resection in total knee arthroplasty

**DOI:** 10.1371/journal.pone.0209317

**Published:** 2018-12-20

**Authors:** Takaaki Ohmori, Tamon Kabata, Yoshitomo Kajino, Daisuke Inoue, Tadashi Taga, Takashi Yamamoto, Tomoharu Takagi, Junya Yoshitani, Takuro Ueno, Ken Ueoka, Hiroyuki Tsuchiya

**Affiliations:** Department of Orthopaedic Surgery, Kanazawa University Hospital Takaramachi13-1 Kanazawa Ishikawa Japan; Universidade Federal Fluminense, BRAZIL

## Abstract

**Purpose:**

During total knee arthroplasty, few rotating reference axes can be reliably used after tibial resection. We speculated that a line that passes through the lateral edge of the posterior cruciate ligament (PCL) at its tibial attachment after resection and the most prominent point of the tibial tubercle [after-tibial resection (ATR) line] will provide a good reference axis. In this study, we aimed to evaluate the association between ATR and Akagi’s lines.

**Materials and methods:**

In this case–control simulation study, we retrospectively evaluated 38 patients with varus knee and 28 patients with valgus knee. We defined the reference cutting plane as 10 mm distal from the lateral articular surface of the tibia in varus group and as 7 mm distal from the medial articular surface in the valgus group. We measured angles between Akagi’s line and the ATR line (ATR line angle) as well as between Akagi’s line and 1/3 Akagi’s line (1/3 Akagi’s line angle), which passes through the midpoint of PCL and the medial third of the patellar tendon. We used paired t-tests to determine the significance of differences between these angles, with p < 0.05 indicating statistical significance. Intra- and interclass correlation coefficients for the reproducibility of 1/3 Akagi’s line angle and ATR line angle were analyzed by two surgeons.

**Results:**

We found that 1/3 Akagi’s line angle was 10.2° ± 1.3° in the varus group and 10.9° ± 1.3° in the valgus group (*p* = 0.017). The ATR line was positioned externally compared with Akagi’s line in all patients. Mean ATR line angles at 0°, 3° and 7° posterior slopes were 6.1° ± 1.9°, 5.8° ± 2.0° and 6.0° ± 1.7° in the varus group and 6.3° ± 2.3°, 6.2° ± 2.3° and 5.4° ± 2.1° in the valgus group, respectively. There were no significant differences in the ATR line angle between the varus and valgus groups. (*p* = 0.34–0.67) Intra- and interclass correlation coefficients for the reproducibility of 1/3 Akagi’s line angle were 0.936 and 0.986 and those for the reproducibility of ATR line angle were 0.811 and 0.839.

**Conclusions:**

The ATR line was positioned between Akagi’s line and 1/3 Akagi’s line in all patients and was a valid option for evaluating rotational tibial alignment after tibial resection.

## Introduction

Implant malalignment in total knee arthroplasty (TKA) is one of the reasons for lower satisfaction with this procedure than with total hip arthroplasty [1]. Malrotation of the tibial component occurs more frequently in TKA and leads to more serious outcomes than malrotation of the femoral component [2]. Several studies have reported internal rotational errors of the tibial component leading to postoperative pain, stiffness and patellofemoral problems [3,4]. To avoid such rotational errors, it is important to have an effective reference axis to facilitate alignment during the procedure.

Many rotating reference axes for the tibial component have been proposed. Akagi’s line is one of the most widely used reference axes [5–8]. This line passes through the midpoint of the posterior cruciate ligament (PCL) and the medial edge of the patellar tendon at their respective tibial attachments; in a normal knee without osteoarthritis, this line runs perpendicular to the surgical epicondylar axis of the knee in the extension position [6,7]. Nevertheless, Akagi’s line needs to be identified before tibial resection, and it cannot be correctly found after resection because the PCL attachment point of the tibia is positioned more outside after tibial resection. There are few established rotating reference axes for the tibial component that can be used after tibial resection. We therefore sought to identify a new reference axis for use after tibial resection by focussing on the lateral edge of PCL at its tibial attachment after resection and on the tibial tubercle, which is the most representative anatomical landmark, as indicated by Matziolis et al. [9]. We hypothesised that after tibial resection, the line passing through the lateral edge of PCL at its tibial attachment and the most prominent point of the tibial tubercle [after-tibial resection (ATR) line] will have slight external rotation from Akagi’s line and that this new line will internally change with increase in the slope of tibial resection. In this study, we aimed to evaluate the association between ATR and Akagi’s lines using virtual cutting with three-dimensional (3D) templating software.

## Materials and methods

### Design and patients

**Fig 1:**
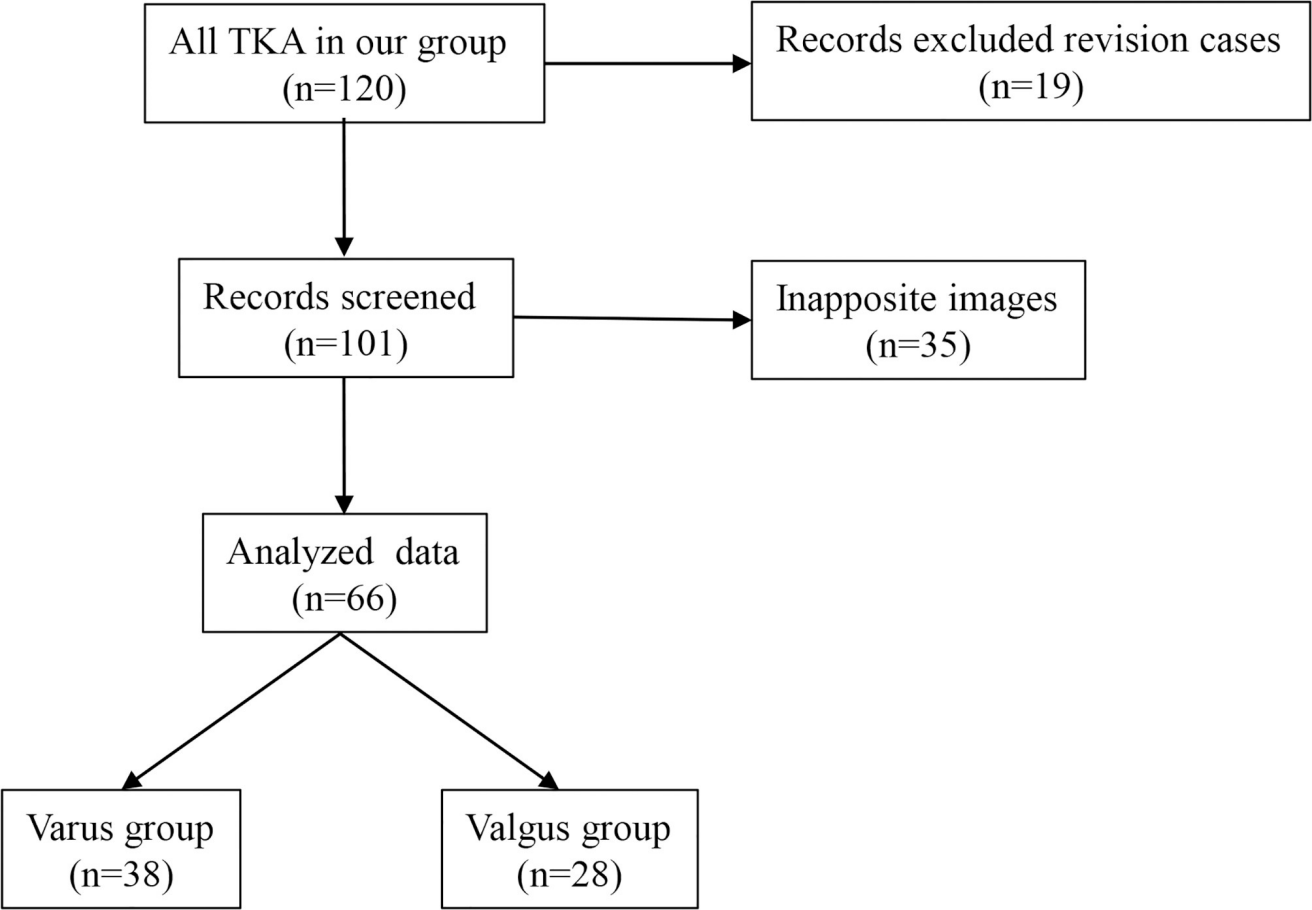
Flowchart of patient selection.

This retrospective, case–control simulation study included 120 patients who had undergone TKA by our group between 2013 and 2016. We excluded revision patients, patients with a history of surgery on the lower leg and patients with inapposite images. Finally, we evaluated 38 patients with varus knee and 28 with valgus knee (Fig 1). Table 1 presents the characteristics of all patients.

**Table 1:** Patient’s detailed information.

	All patients (n = 66) (male = 13, female = 53)		
	Varus group (n = 38)	Valgus group (n = 28)	P value
Age (years)	73.4 ± 6.8	69.8 ± 9.0	0.068
Gender	Male: 7, female: 31	Male: 6, female: 22	0.765
BMI (kg/m^2^)	26.7 ± 4.1	25.0 ± 4.5	0.106
HKA (Hip knee ankle) angle	10.0 ± 4.9° (varus)	11.7 ± 7.4° (valgus)	<0.001

Patients provided informed consent for the use of their data in this publication. All procedures were approved by the Ethical Committee of the Graduate School of Medical Sciences, Kanazawa University (approval #1751).

### Anatomical model and reference planes

We constructed a 3D model of the tibia based on computed tomography (CT) images (LightSpeed VCT Series/Discovery CT750; GE Healthcare, Tokyo, Japan). We transferred imaging data to a CT-based 3D templating system (ZedKnee, Lexi Co., Ltd., Tokyo, Japan).

**Fig 2:**
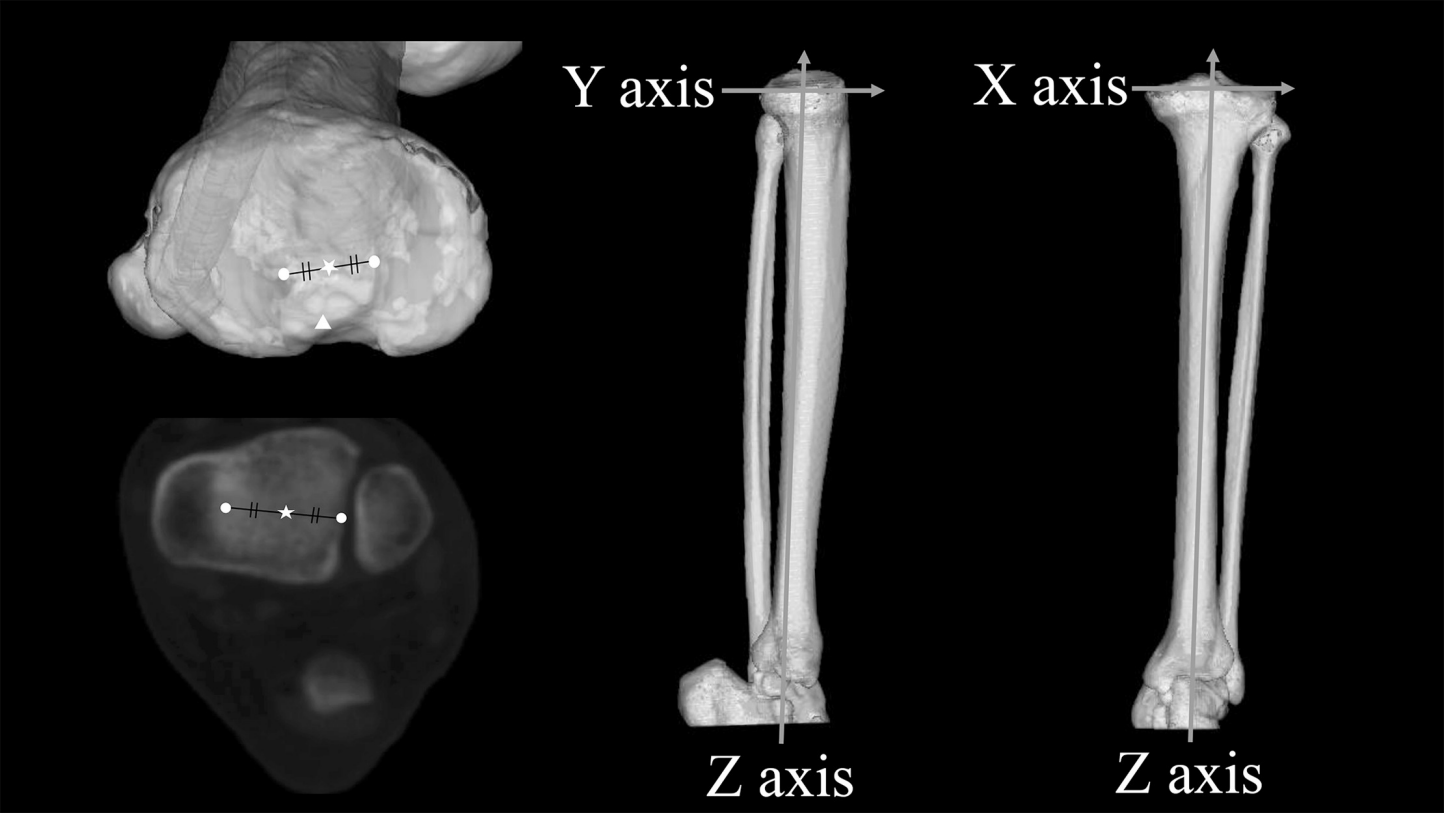
Definitions of the axes in the tibial reference plane. Left Upper: Tibial surface before resection 〇: Medial and lateral eminentia intercondylaris ☆: Centre of the medial and lateral eminentia intercondylaris △: Midpoint of the posterior cruciate ligament Lower: 2D plane of the ankle joint 〇: Medial and lateral edge of the ankle joint ☆: Centre of the ankle joint Middle: YZ plane The Z-axis was defined as the line connecting the centre of the medial and lateral eminentia intercondylaris and the centre of the ankle. The Y-axis was defined as the line perpendicular to the Z-axis from the midpoint of the PCL. Right: XZ plane The X-axis was defined as the line perpendicular to the YZ plane bisecting the Y-axis.

We defined a coordinate system for the tibia for use with all measurements and simulations. We defined the *Z*-axis as the line connecting the centre of the medial and lateral eminentia intercondylaris and the centre of the ankle; the *Y*-axis as the line perpendicular to the *Z*-axis from the midpoint of the PCL and the *X*-axis as the line perpendicular to the YZ plane and bisecting the *Y*-axis (Fig 2).

### Cutting plane of the tibia

We defined the reference cutting plane as 10 mm distal from the tibial lateral articular surface in the varus group and as 7 mm distal from the tibial medial articular surface in the valgus group. As previous studies have shown the cartilage to be approximately 2 mm thick [10] and as we were unable to identify the cartilage on CT images, we defined the cutting line as 8 mm below the deepest point of the lateral articular surface in the varus group and as 5 mm below the deepest point of the medial articular surface in the valgus group. The posterior slope of the cutting plane was changed to 0°, 3° or 7°.

### Rotating reference axes

**Fig 3:**
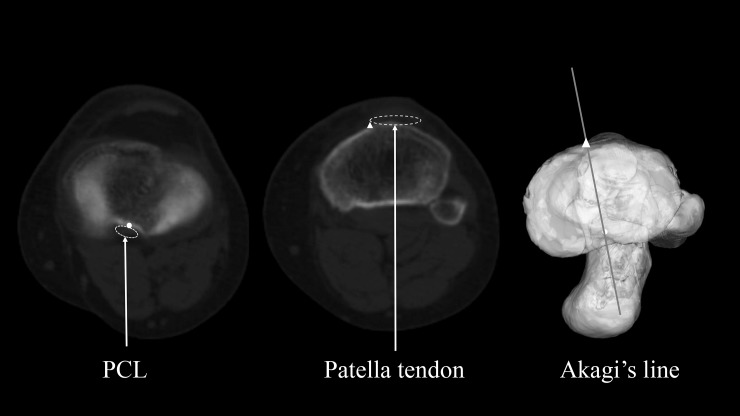
Definition of Akagi’s line. Left (〇): Midpoint of the PCL at the tibial attachment before resection Middle (△): Medial edge of the patellar tendon at the tibial attachment Right: Akagi’s line on 3D model.

**Fig 4:**
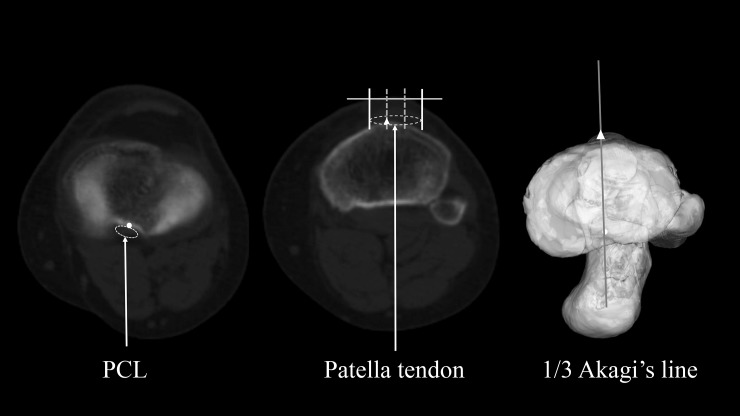
Definition of 1/3 Akagi’s line. Left (〇): Midpoint of the PCL at the tibial attachment before resection Middle (△): Medial third of the patellar tendon at the tibial attachment Right: 1/3 Akagi’s line on 3D model.

**Fig 5:**
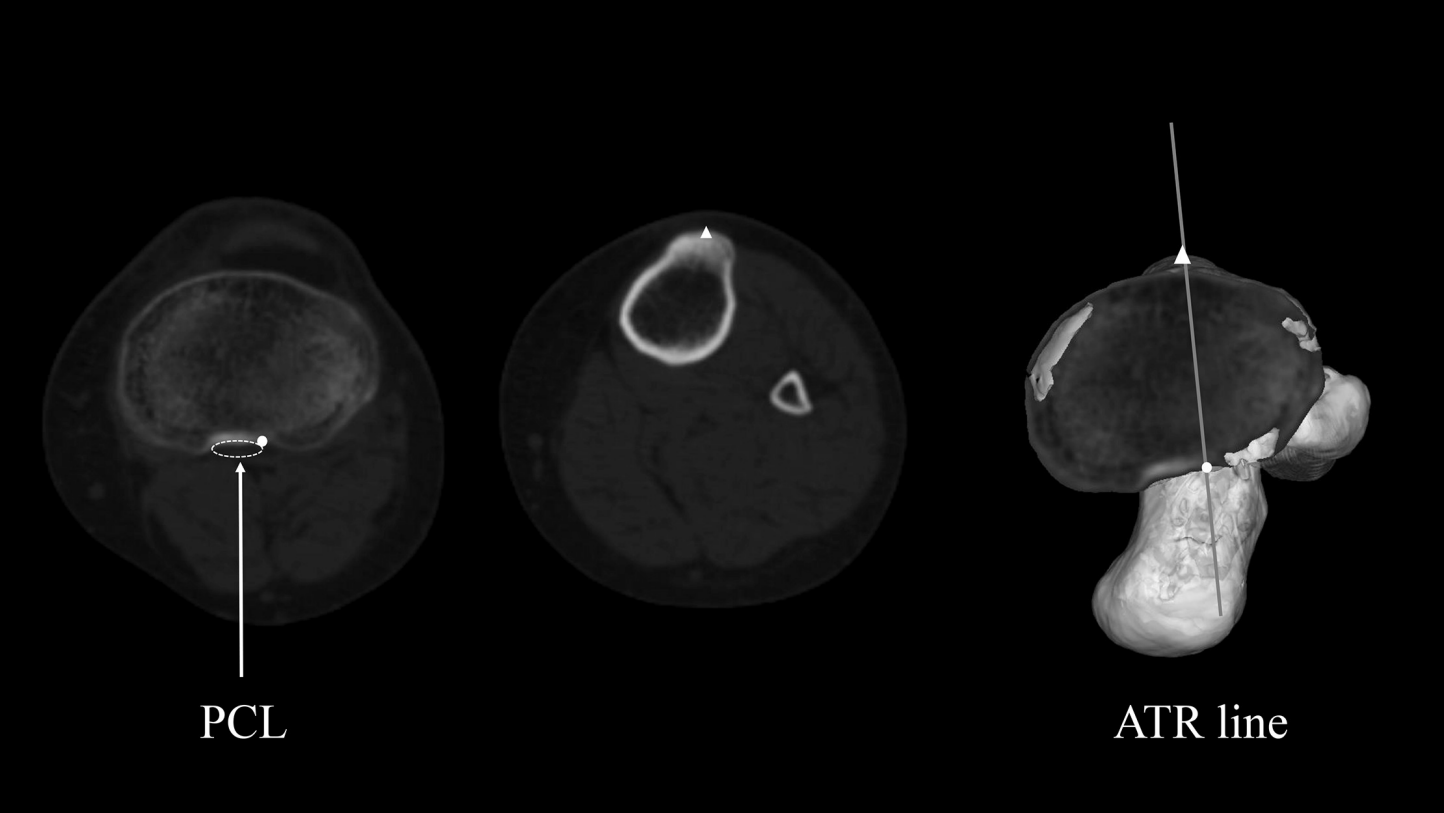
Definition of the ATR line. Left (〇): The lateral edge of the PCL at the tibial attachment after resection Middle (△): The most prominent point of the tibial tubercle Right: The ATR line on 3D model.

**Fig 6:**
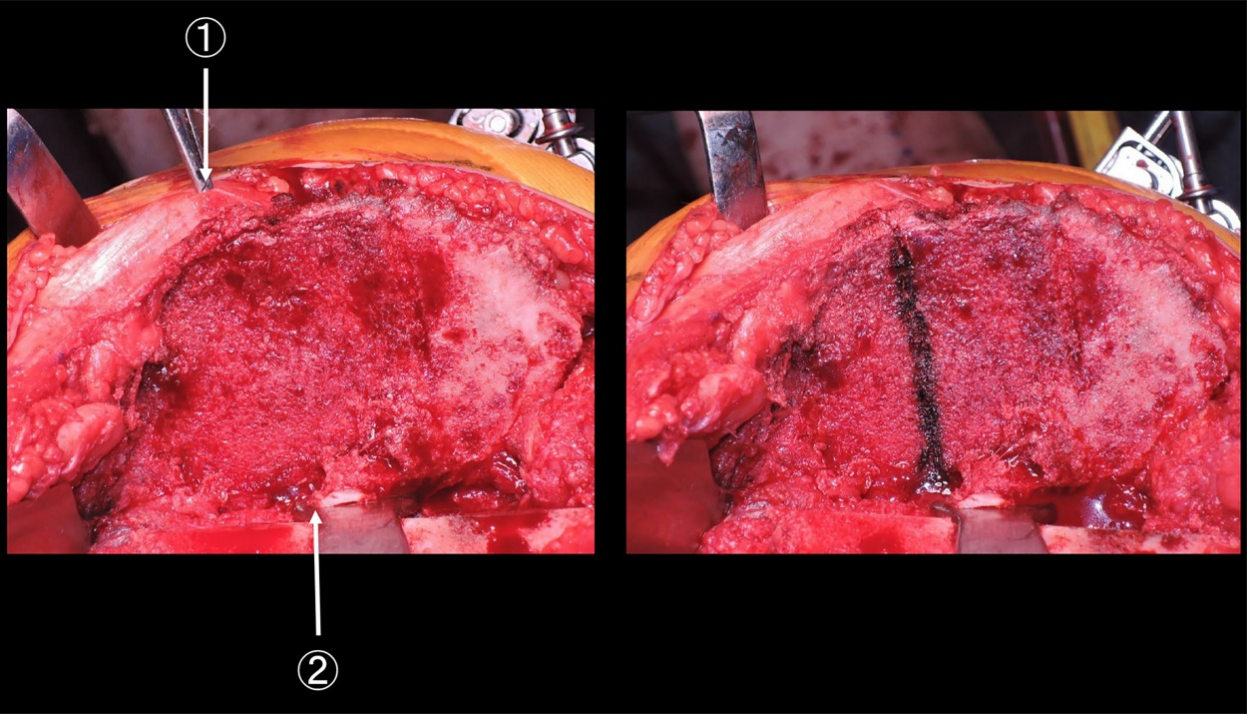
Clinical photo of the ATR line. Left: The tibial surface after osteotomy ①: The most prominent point of the tibial tubercle ②: The lateral edge of the PCL at the tibial attachment Right: The ATR line.

As previously mentioned, Akagi’s line passes through the midpoint of PCL at the height of the original joint surface and the medial edge of the patellar tendon at their respective tibial attachments (Fig 3) [5–8]. We defined 1/3 Akagi’s line as the line passing through the midpoint of PCL at the height of the original joint surface and the medial third of the patellar tendon at their respective tibial attachments (Fig 4) [8,11–13]. We defined the ATR line as the line passing through the lateral edge of PCL at its tibial attachment after tibial resection and the prominent point of the tibial tubercle (Figs 5 and 6). Those two points were easily recognised by directly looking down the resection plane.

### Measurements

**Fig 7:**
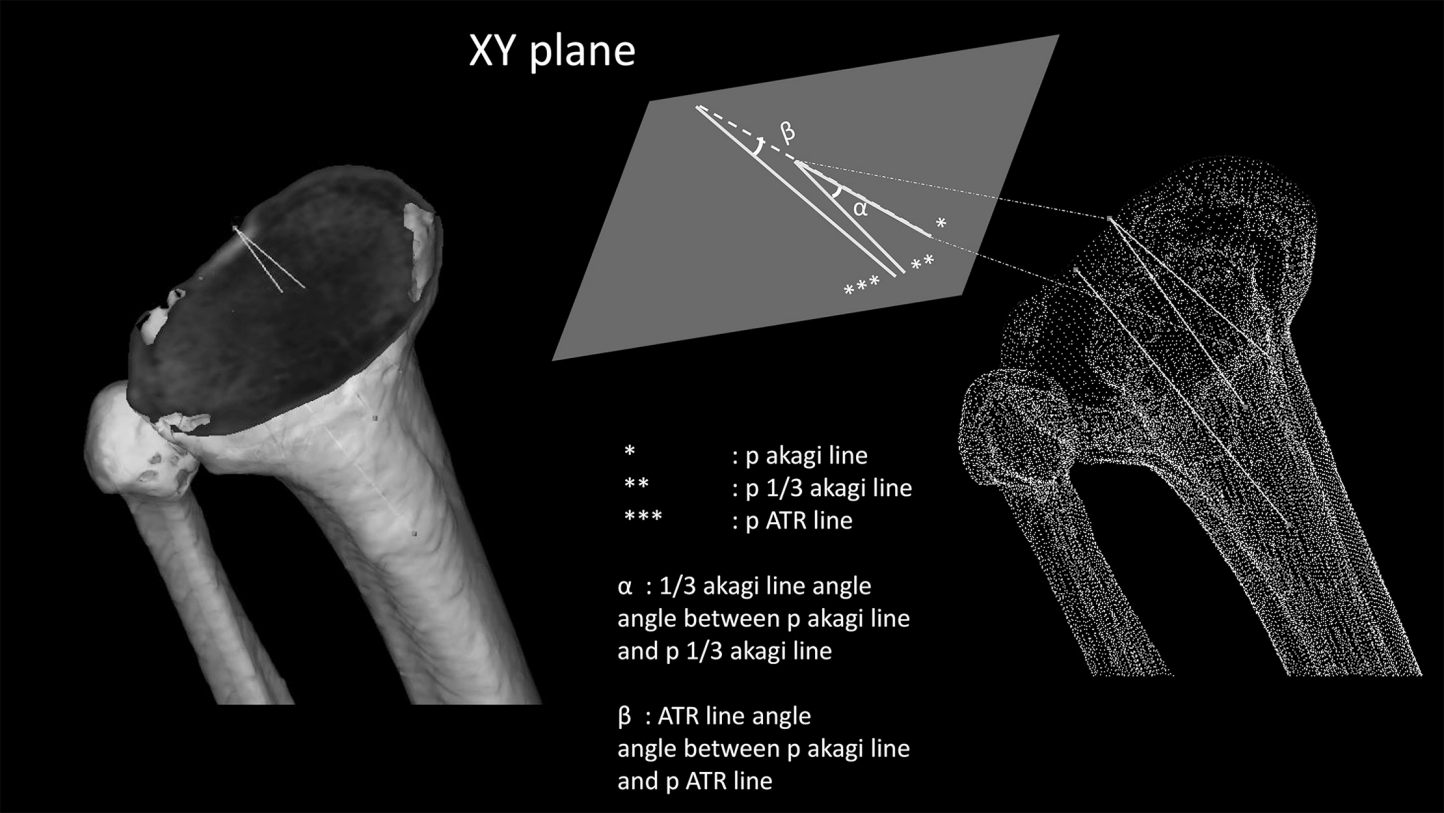
The three lines and their projections (p) on the tibial XY plane. Left: Akagi’s line, 1/3 Akagi’s line and the ATR line in the 3D model Right: Definitions of ‘p Akagi’s line’, ‘p 1/3 Akagi’s line’ and ‘p ATR line’.

We projected these three lines onto the XY plane of the tibia, naming these as ‘p Akagi’s line’, ‘p 1/3 Akagi’s line’ and ‘p ATR line’ (Fig 7). We measured 1/3 Akagi’s line angle, which was defined as the angle between p 1/3 Akagi’s line and p Akagi’s line, and the ATR line angle, which was defined as the angle between p ATR and p Akagi’s lines. We then evaluated the association between these two angles. Positive values are defined as external rotation from Akagi’s line.

### Statistical analysis

One surgeon measured the data for all patients twice, at least 2 weeks apart. We used the mean of the two measured values for analysis. Another surgeon measured the data of 20 patients who were randomly chosen among all patients to evaluate the intraobserver error. We expressed all data as mean and standard deviation and analysed differences using SPSS statistical software version 23 (IBM Corp., Armonk, NY, USA). We used paired t-tests to determine the significance of differences in 1/3 Akagi’s line angle and ATR line angle between the valgus and varus groups, with *p* < 0.05 indicating statistical significance.

### Power analysis

Based on a prior power analysis, we set an alpha level of 0.05 and a power of 0.80. Since the standard deviation for each line was about 2° in our preliminary survey. We considered the difference of at least 1.5° or more to be meaningful. Thus, after setting the effect size of 0.80, the sample size was calculated to be 52 (26 in each group).

## Results

Intra- and interclass correlation coefficients for the reproducibility of 1/3 Akagi’s line angle were 0.936 and 0.986 and those for the reproducibility of ATR line angle were 0.811 and 0.839 (Table 2).

**Table 2:** Inter- and intra- observer correlation.

	1/3 Akagi's line angle	ATR line angle
inter-observer correlation	0.986	0.811
intra-observer correlation	0.936	0.839

**Fig 8:**
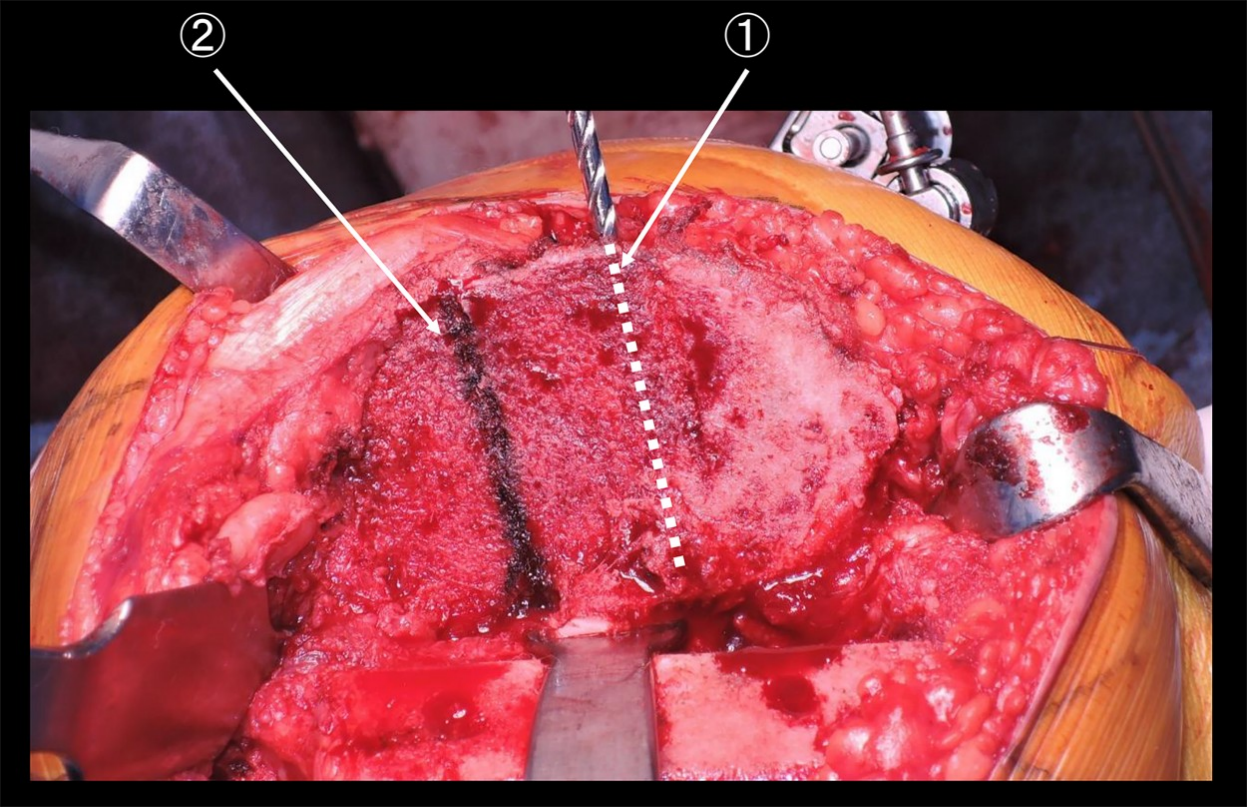
Relationship between Akagi’s line and the ATR line. ①: The line parallel to Akagi’s line ②: The ATR line.

We found that 1/3 Akagi’s line angle was 10.2° ± 1.3° (range: 7.8°–12.3°) in the varus group and 10.9° ± 1.3° (range: 7.6°–14.4°) in the valgus group (*p* = 0.017). The ATR line was positioned externally compared with Akagi’s line and internally compared with 1/3 Akagi’s line in all patients. In other words, the ATR line was positioned between Akagi’s line and 1/3 Akagi’s line in all patients. Fig 8 showed clinical photo. This represents the plane after the tibial resection. The ATR line was positioned externally compared with Akagi’s line.

Table 3 shows changes in the ATR line angle with changes in posterior inclination slopes of tibial resection. Mean ATR line angles at with 0°, 3° and 7° posterior slopes were 6.1° ± 1.9°, 5.8° ± 2.0° and 6.0° ± 1.7° in the varus group and 6.3° ± 2.3°, 6.2° ± 2.3° and 5.4° ± 2.1° in the valgus group, respectively. There were no significant differences in the ATR line angle between the varus and valgus groups. (*p* = 0.34–0.67)

**Table 3:** 1/3 akagi’s line angle and ATR line angle of each group.

		Varus group (n = 38)	Valgus group (n = 28)	Mean difference (95% CI)	P value
1/3 akagi’s line angle (°)		10.2 ± 1.3 (range 7.8–12.3)	10.9 ± 1.3 (range 7.6–14.4)	-0.803 (-1.46 to -0.148)	0.017
	0°	6.1 ± 1.9	6.3 ± 2.3	-0.226 (-1.28 to 0.827)	0.67
ATR line angle (°)	3°	5.8 ± 2.0	6.2 ± 2.3	-0.376 (-1.45 to 0.699)	0.49
	7°	6.0 ± 1.7	5.4 ± 2.1	0.472 (-0.502 to 1.45)	0.34

## Discussion

The most important finding of the present study was that the ATR line is positioned between Akagi’s line and 1/3 Akagi’s line. Some studies have described the configuration of the surface geometry of the tibia. Ushio et al. [14] have reported that after tibial resection, the anteroposterior axis, which is defined as the perpendicular bisector of the medial and lateral condylar centres, was rotated internally compared to that before resection. Alternatively, anteroposterior axis based on the surface shape may be internally rotated after tibial resection. Forster-Horvath et al. [15] have reported similar observations regarding tibial morphology. Thus, if a surgeon positions the tibial component in accordance with the tibial surface geometry, it would be rotated internally. Some researchers have noted that contemporary tibial component designs do not match the morphology of the tibia [16,17]. The tibial attachment of PCL to the cutting plane moves laterally compared to the attachment of PCL to the artificial surface; hence, the line between the midpoint of PCL and the medial edge of the patellar tendon at their respective tibial attachments would be rotated internally after resection compared to the original Akagi’s line. Thus, the geometry of the tibial cutting plane can lead to many pitfalls that can subsequently result in internal malalignment of the tibial component. Previous studies have reported that the tibial component is likely to be positioned more internally than the ideal setting [18–20]. In addition, few reports have indicated the index of tibial rotation axis on tibial resection plane. Therefore, we believe that some indexes of tibial rotational axis on tibial resection plane is important. Kawahara et al. [18] have reported that the line passing through the geometric centre of the resection plane and the medial sixth of the patellar tendon at the tibial attachment is close to Akagi’s line and that it can be used as a rotating reference axis after tibial resection. We believe that a greater distance between reference points is better for reducing measurement errors. Therefore, we proposed a new anatomic rotating reference axis for the tibia after resection—the ATR line.

Akagi’s line is one of the most widely used reference axes and has been reported to be the most reliable by Saffarini et al. [8]. In addition, other rotating reference axes of the tibia, such as the midtransverse axis, anteroposterior axis, Berger line [21], midsulcus line [22], posterior condylar line of the tibia [23] and transcondylar line of the tibia [24], have been reported. Another proposed axis is the line passing through the centre of the tibial resection plane and the medial third of the tibial tubercle [22]. Rossi et al. [25] have recommended the use of posterolateral corner locked technique, in which the tibial component is rotated until its anteromedial border corresponds with the anteromedial margin of the tibia, ensuring that the posterolateral corner of the tibial component matches the posterolateral corner of the tibia. However, this technique may lack accuracy for patients with osteophytes or bone defects.

Akagi et al. evaluated Akagi’s line with patients in the knee extension position [6]. Li et al. [26] have reported that knee alignment changed during weight-bearing activity and that rotational alignment moved towards a maximum of 10° external rotation when the knee position changed from extension to flexion. Notably, rotational mismatch would be prevented when the tibial component is placed more externally than Akagi’s line. Sanford et al. [27] and Lawrie et al. [28] have reported similar suggestions on changing rotational alignment. Steinbrücker et al. [29] have reported that the internal rotation of the tibial component led to higher retropatellar pressure than external rotation and that this influenced postoperative pain. They recommended rotational alignment of the tibial component to the line passing through the medial third of the tibial tubercle. In the present study, 1/3 Akagi’s line was rotated approximately 10° externally to Akagi’s line, which is consistent with past reports. Based on the above results, we think that it is important to place the tibial component at rotational alignment between Akagi’s line and 1/3 Akagi’s line.

The ATR line was positioned between Akagi’s line and 1/3 Akagi’s line in all patients. In addition, the ATR angle was almost half the 1/3 Akagi’s line angle. Tibial rotating reference axes have been proven to be controversial [30]. Therefore, we assume that the ATR line is a useful option for many orthopaedic surgeons. In addition, there were no significant differences between the varus and valgus groups, indicating that the reference points of the ATR line are good anatomical landmarks that are not affected by varus and valgus deformities. Moreover, the ATR line was not affected by the tibial posterior slope, indicating that it can be used regardless of posterior inclination.

There are four limitations in this study. First, this was simulation study; hence, we could not establish the accuracy of the technique in clinical practice. Second, the cutting line was fixed at distal from the lateral or medial artificial surface on CT. Changing the cutting level may change the ATR line, although this also applies to other reference lines. Third, if PCL has bony islands, it may be hard to recognise the lateral edge of the PCL attachment. Fourth, there are no clinical outcome data with the use of the ATR line as a reference axis. In the future, it is necessary to investigate clinical outcomes. Despite these limitations, the ATR line may be a good option for confirming the rotation axis after tibial bone cutting, and it may be useful index for orthopaedic surgeons.

There are few rotating reference axes for tibial components, and it is necessary to use maximum references to prevent misalignment. Our findings demonstrate that the ATR line is a useful rotating reference axis, which is unaffected by the posterior slope after tibial resection.

## Conclusions

In summary, the ATR line is positioned between Akagi’s line and 1/3 Akagi’s line and can be a valid option for evaluating rotational tibial alignment after tibial resection.

## Supporting information

S1 Checklist Strobe checklist. (PDF)
